# Interleukin 8 (-251 T>A) polymorphism in children and teenagers infected with *Helicobacter pylori*

**DOI:** 10.1186/s40409-017-0113-z

**Published:** 2017-04-08

**Authors:** Marina Saes, Roger Willian de Labio, Lucas Trevizani Rasmussen, Spencer Luiz Marques Payão

**Affiliations:** 1 Faculdade de Medicina de Marília (FAMEMA), rua Lourival Freire, 240, Jardim Fragata, CEP 17519-050 Marília, SP Brazil; 2 Universidade do Sagrado Coração, Bauru, SP Brazil

**Keywords:** *Helicobacter pylori*, Interleukin 8, Polymorphism, -251 (T>A), Gastric disease

## Abstract

**Background:**

*Helicobacter pylori* (*H. pylori*) is a gram-negative bacterium that colonizes the human stomach and causes a variety of gastric diseases. This study evaluated the correlations between the -251 (T>A) (rs4073) polymorphism of interleukin-8 (IL-8), the etiology of gastric disease, and *H. pylori* infection in pediatric and adolescent patients.

**Methods:**

DNA samples were obtained from 285 gastric biopsies from pediatric patients. *H. pylori* was detected by PCR, whereas PCR-RFLP was used to characterize the -251 (T>A) polymorphism of IL-8.

**Results:**

The histological analysis revealed the presence of gastritis in 158 patients (55.44%). *H. pylori* was found in 71 samples (24.9%). The -251 (T>A) polymorphism revealed that 58 (29.47%) samples were TT, 143 (50.18%) samples were TA, and 84 (20.35%) samples were AA.

**Conclusions:**

Our findings suggest that IL8-251 A allele may be an important risk factor for the development of gastric disease when associated with *H. pylori* infection.

## Background

*H. pylori* is a gram-negative bacterium that colonizes the antrum and/or the body of the human stomach, causing complications such as gastritis and gastric ulcers, duodenal ulcers, MALT lymphoma, and a few types of gastric adenocarcinoma [1, 2]. *H. pylori* is present in approximately half of the world’s population. The outcome of the infection is determined by its duration, as well as by environmental, host-related and bacterial factors [3, 4].

The symptoms of the gastric diseases caused by *H. pylori* occur most frequently in adults, though the bacterium may have been acquired in childhood. Several routes of infection have been described, but the oral ingestion of bacteria and transmission between family members in childhood are the two most likely ways in which the infection is transmitted in childhood [5–8].

After infection, *H. pylori* accesses the gastric mucosa and triggers the production of cytokines that promote recruitment of inflammatory cells, which are likely involved in tissue damage. Chronic inflammation produces changes to gastric morphology, prevents apoptosis, and causes angiogenesis, thus leading to neoplastic lesions and cancer [9].

The association between genetic factors of the bacterium and those of the host may influence the host response to infection. When it comes to the genetic factors of the host, interleukins are of particular importance. Interleukins are peptide molecules that mediate interactions between cells of the immune system and other systems, including the endocrine system. According to Kamali-Sarvestani et al. [10], the IL-8 gene is one of the most important candidate host genes in determining the outcome of *H. pylori* infection.

A previous study has suggested that IL-8 production is genetically determined and that individuals who are homozygous for the AA genotype at the -251 position demonstrate a trend toward higher levels of IL-8 production [11]. Two other studies reported that patients with the IL-8-251 TA and AA genotypes are more susceptible to *H. pylori* infection and gastric cancer [12, 13].

In light of previous findings that gene polymorphism of proinflammatory cytokines are important genetic factors of the host in *H. pylori* infection and that acquisition may have occurred during childhood, this study seeks to assess whether the -251 (T>A) polymorphism of IL-8 is correlated with the etiology of gastric disease and/or with *H. pylori* infection in pediatric and adolescent patients in Brazil.

DNA samples were obtained from 285 gastric biopsies of pediatric and adolescent patients ranging from 1 to 14 years of age (116 ♂/169 ♀, mean age ♂ 7.77 ± 3.5 years; mean age ♀ 9.28 ± 3.29 years) with gastric symptoms. All patients were recruited from the Endoscopy Department of Marília Medical School in the state of São Paulo, Brazil.

Two biopsies were collected from each patient. The first fragment was used in the histopathological analysis according to the Sydney system [14], while the second fragment was used in the molecular analysis. All subjects who had received antimicrobial therapy and anti-inflammatory agents or proton pump inhibitors at least 30 days prior to endoscopy were excluded.

Genomic DNA was extracted from the gastric biopsies according to the protocol established by the manufacturer of the QIAamp DNA Mini kit®, cat. no. 51306 (Qiagen, Germany). *H. pylori* was detected using PCR in accordance with previous studies [15].

Genotypes were determined using the polymerase chain reaction restriction fragment length polymorphism (PCR-RFLP) method described by Hamajima et al. [16]. *Helicobacter pylori* was detected by PCR in 71 samples. The histological analysis diagnosed the presence of gastritis in 158 patients (55.44%), and 61 of these were found to be infected by *H. pylori* (Table 1). The characterization of the -251 (T>A) polymorphism is described in Table 2.

**Table 1:** Association between the presence of *H. pylori* and changes in the gastric mucosa

	*H. pylori* Negative	*H. pylori* Positive
Normal mucosa	117 (92%)	10 (8%)
Gastritis	97 (61%)	61 (39%)
Total	214 (75%)	71 (25%)

**Table 2:** Allele and genotype distribution of interleukin 8, -251 (T>A) polymorphism

Distribution of IL-8 alleles in the 285 histologically analyzed samples						
	Total		Normal mucosa		Gastritis	
T	311	55%	114	57%	167	53%
A	259	45%	110	43%	149	47%
Total	570	100%	224	100%	316	100%
Distribution of IL-8 genotypes in the 285 histologically analyzed samples						
	Total		Normal mucosa		Gastritis	
A/A	58	20%	26	20%	32	20%
T/A	143	50%	58	46%	85	54%
T/T	84	30%	43	34%	41	26%
Total	285	100%	127	100%	158	100%

There were no statistically significant differences between the IL-8 genotypes of patients with gastritis and patients with normal mucosa. However, when all individuals infected with *H. pylori* were considered, the AA genotype was found to have a higher chance of developing gastritis (OR = 16.30; CI = 1.99 to 133.58) when compared to the TA and TT genotypes.

Other studies have reported consistent findings; the increase in IL-8 production has been found to be associated with the presence of the A allele [7, 17]. IL-8 production is increased by the presence of the A allele, and the quality and intensity of inflammatory responses produced by the host may be altered after exposure to *H. pylori* [5, 7, 17]. Ohyauchi et al. [13] and Taguchi et al. [18] found that the AA genotype produces a higher risk of atrophic gastritis than the TT genotype does. The authors also associated AA and TA genotypes with higher levels of IL8 and with a greater degree of neutrophil infiltration when comparing these genotypes with the TT genotype. Hofner et al. [19] described an association between the IL-8 (TA) genotype and risk of gastritis or duodenal ulcers in patients infected with *H. pylori*.

However, other studies have reported conflicting results. Cheng et al. [20] found no association between the IL8-251 T>A polymorphism and increased risk of gastritis in Thai patients. Fabris et al. [21] also failed to find any significant association between the IL-8-251 T>A polymorphism and *H. pylori* infection in Brazilian patients. However, Caleman Neto et al. [15] studied adult gastric patients in Brazil and found a significant difference between the TA and TT genotypes in terms of *H. pylori* presence; however, no correlation was found between the AA genotype and the occurrence of *H. pylori*. Their results suggest that the presence of the TA genotype may be a risk factor for *H. pylori* infection whereas the TT genotype may act as a protective factor against *H. pylori*. Our study on pediatric patients, nevertheless, did not reveal any statistically significant variation between the different IL-8-251 polymorphism genotypes and changes in gastric epithelia, though individuals with the AA genotype who tested positive for *H. pylori* did have a greater chance of developing gastritis (OR = 16.30/1.99 to 133.58).

Chongruksut et al. [22] reviewed updated basic research studies on links between inflammatory cytokine genetic expression level and gastric cancer (GC) risk in a Thai population. The review focused on IL-8 mRNA expression and *Helicobacter pylori* infection, and an increased risk of GC and aggressive histologic types was found. The authors performed an in-depth analysis of the epidemiological data and determined various cut-off points in order to discern which level of IL-8 mRNA expression was able to predict GC occurrence.

Useful future studies may analyze IL-8 polymorphisms and expression levels in different stages of gastritis in both pediatric and adult patients in order to determine whether IL-8 is more sensitive than *H. pylori* in cases of gastric disease.

The results revealed an association between the presence of gastric disease and infection with *H. pylori*. Our findings also suggest that IL8-251 A allele may be an important risk factor for the development of gastric disease when associated with *H. pylori* infection in pediatric patients.

## Abbreviations

GC: Gastric cancer
IL-8: interleukin-8

## References

[1] Warren R, Marshall BJ (1983). Unidentified curved bacilli on gastric epithelium in active chronic gastritis. Lancet.

[2] Kusters JG, van Vliet AHM, Kuipers EJ (2006). Pathogenesis of *Helicobacter pylori* infection. Clin Microbiol Rev.

[3] Mitchell HM (1999). The epidemiology of *Helicobacter pylori*. Curr Top Microbiol Immunol.

[4] Zabaglia LM, Ferraz MA, Pereira WN, Orcini WA, de Labio RW, Neto AC (2015). Lack of association among *TNF*-alpha gene expression, -308 polymorphism (G > A) and virulence markers of *Helicobacter pylori*. J Venom Anim Toxins Incl Trop Dis.

[5] Bonamico M, Mariani P, Magliocca FM, Petrozza V, Montuori M, Pezzella C (1997). *Helicobacter pylori* duodenal colonization in children. Acta Paediatr.

[6] Rowland M, Kumar D, Daly L, O’Connor P, Vaughan D, Drumm B (1999). Low rates of *Helicobacter pylori* reinfection in children. Gastroenterology.

[7] Lehours P, Yilmaz O (2007). Epidemiology of *Helicobacter pylori* infection. Helicobacter.

[8] Kivi M, Rodin S, Kupershmidt I, Lundin A, Tindberg Y, Granstrom M (2007). *Helicobacter pylori* genome variability in a framework of familial transmission. BMC Microbiol.

[9] Zabaleta J (2012). Multifactorial etiology of gastric cancer. Methods Mol Biol.

[10] Kamali-Sarvestani E, Bazargani A, Masoudian M, Lankarani K, Taghavi AR, Saberifiroozi M (2006). Association of *H pylori cag*A and *vac*A genotypes and IL-8 gene polymorphisms with clinical outcome of infection in Iranian patients with gastrointestinal diseases. World J Gastroenterol.

[11] Hull J, Ackerman H, Isles K, Usen S, Pinder M, Thomson A (2001). Unusual haplotypic structure of IL8, a susceptibility *locus* for a common respiratory virus. Am J Hum Genet.

[12] Lu H, Xiao SD (2014). New ideas for future studies of *Helicobacter pylori*. J Dig Dis.

[13] Ohyauchi M, Imatani A, Yonechi M, Asano N, Miura A, Iijima K (2005). The polymorphism interleukin 8-251 A/T influences the susceptibility of *Helicobacter pylori* related gastric diseases in the Japanese population. Gut.

[14] Stolte M, Meining A (2001). The updated Sydney system: classification and grading of gastritis as the basis of diagnosis and treatment. Can J Gastroenterol.

[15] Caleman Neto A, Rasmussen LT, de Labio RW, de Queiroz VF, Smith MAC, Viani GA (2014). Gene polymorphism of interleukin 1 and 8 in chronic gastritis patients infected with *Helicobacter pylori*. J Venom Anim Toxins Incl Trop Dis.

[16] Hamajima N, Katsuda N, Matsuo K, Saito T, Hirose K, Inoue M (2003). High anti-*Helicobacter pylori* antibody seropositivity associated with the combination of IL-8-251TT and IL-10-819TT genotypes. Helicobacter.

[17] Ye BD, Kim SG, Park JH, Kim JS, Jung HC, Song IS (2009). The interleukin-8-251 A allele is associated with increased risk of noncardia gastric adenocarcinoma in *Helicobacter pylori*-infected Koreans. J Clin Gastroenterol.

[18] Taguchi A, Ohmiya N, Shirai K, Mabuchi N, Itoh A, Hirooka Y (2005). Interleukin-8 promoter polymorphism increases the risk of atrophic gastritis and gastric cancer in Japan. Cancer Epidemiol Biomarkers Prev.

[19] Hofner P, Gyulai Z, Kiss ZF, Tiszai A, Tiszlavicz L, Toth G (2007). Genetic polymorphisms of NOD1 and IL-8, but not polymorphisms of TLR4 genes, are associated with *Helicobacter pylori*-induced duodenal ulcer and gastritis. Helicobacter.

[20] Cheng HH, Chang CS, Wang HJ, Wang WC (2010). Interleukin-1beta and -10 polymorphisms influence erosive reflux esophagitis and gastritis in Taiwanese patients. J Gastroenterol Hepatol.

[21] Fabris RC, Rasmussen LT, Neto AC, de Labio RW, Orcini W, Ximenez JPB (2012). Polimorfismo da Interleucina-8 -251 T>A e *Helicobacter pylori*. ACM.

[22] Chongruksut W, Limpakan Yamada S, Chakrabandhu B, Ruengorn C, Nanta S (2016). Correlation of *Helicobacter pylori* and interleukin-8 mRNA expression in high risk gastric cancer population prediction. World J Gastrointest Oncol.

